# The Effects Induced by a Specific Program on the Development of Segmental Flexibility in Athletes Aged 7–14 in Synchronized Swimming

**DOI:** 10.3390/children9010017

**Published:** 2021-12-28

**Authors:** Adela Badau, Barna Szabo-Csifo, Laura Ciulea, Razvan Alexandrescu, Dana Badau

**Affiliations:** Faculty of Sciences and Letters, “George Emil Palade” University of Medicine, Pharmacy, Sciences and Technology, 540142 Targu Mures, Romania; adela.badau@umfst.ro (A.B.); razvanumf1998@gmail.com (R.A.); dana.badau@umfst.ro (D.B.)

**Keywords:** synchronized swimming, segmental flexibility, sports, training period, age

## Abstract

This research aims to expand the knowledge on the level of development of segmental flexibility, to girls aged 7–14 years, who practice synchronized swimming. The study includes 112 girls aged between 7 and 14 years, divided into groups on age, every two years, and on the period of synchronized swimming between 6 months and 42 months. The study focused on three body segments, namely: torso, hip, and shoulder. Segmental flexibility was assessed using 5 tests: standing trunk flexion, shoulder flexibility, Hip-split legs sideways, Hip-split antero-posterior with the right foot forward, and Hip-split antero-posterior with the left foot forward, performed in the gym. The statistical analysis was performed using the SPSS-24 software aiming at the following parameters: arithmetic means (X), standard deviation (SD), minimum (Min), maximum (Max), CI–95% Confidence Interval for Mean with the two lower and upper marks, Kolmogorov-Smirnov test for testing the normality of data distribution and a multifactor ANOVA analysis, using the F test. The most significant improvements highlighted by the differences between initial and final were for: the shoulder flexibility test in the 13–14 years’ groups; flexibility of the spine registered the biggest difference between the 9–10 years’ group; for hip-split legs sideways the biggest difference was between 9–10 years’ group and 13–14 years and 9–10 years, too. The hip-split antero-posterior tests with the left and also, for right foot forward, showed the biggest differences between tests for 13–14 age groups. The development of joint flexibility has an upward evolution, being conditioned by the age of the practitioners and by the operating methodology specific to synchronized swimming. The longer the training period, the greater the premises for the development of segmental flexibility.

## 1. Introduction

Synchronized swimming is a sport discipline, which requires the development of specific technical elements, called mandatory figures, according to some models adopted, requiring a high performance, within the competition [1,2].

The higher level of technicality specific to competitive synchronous swimming requires the performance of complex and synchronous figures with high parameters of technique, shape, and amplitude and which are based on the special abilities of practitioners in terms of functional physical capacity [3].

The specificity of synchronized swimming involves precise synchronized movements and high-risk acrobatic maneuvers, mostly performed underwater, requiring flexibility, kinesthetic awareness, and aerobic and anaerobic conditioning [4,5].

For the realization with a high technicality of the imposed elements, an important factor is the level of development of the motor qualities. Segmental flexibility is an essential condition that contributes to the increase of spectacularity from the point of view of artistic appreciation, being responsible for the accuracy and precision of the shape of the elements performed, from a technical point of view [2]. Flexibility has a tendency to decrease throughout life, except for the period between 10 and 13 years where it is significantly developed by increasing disproportion, in which the lower limbs become proportionally longer compared to the upper body, avoiding overloads of the musculoskeletal system [6]. Throughout life, flexibility improves only in the direction in which it is intervened [7].

According to FINA, the technical evaluation takes into account Visible scales of deviation of the angle, from 1 to 15 degrees from a small, from 16–30 degrees for a medium, and over 30 degrees for large deviation, which implies a process of continuous training of efficient and concrete segmental flexibility, both at the level of the lower limbs, the hip joint as well as the scapula-humeral center and the spine. The technical evaluation representing 30% of the final grade in different powers depending on the work formation on execution and synchronization, aims at the following aspects: the design representing the degree of compliance with those positions and movements specified in the description of the figure reported to the accuracy of all body positions and transitions; the control manifested by the level of performance achieved by the control factors aiming at the use of force and coordination to demonstrate mastery of figure execution aiming for example: maintains stable correct positions, specific transient body movements accurately and effortlessly, a general impression of ease in achieving performance, the direction of execution of movements, as well as ease and synchronization in performing specific figures [8].

The evaluation of the technical level in synchronized swimming focuses on the appreciation of the level of performance achieved on a series of imposed figures, a level largely influenced by the degree of flexibility and amplitude of the movements. In this sense, a study on flexibility in girls aged 12.7 ± 1.1 years, who practiced swimming, showed that in the training process there is no special emphasis on the development of this quality, concluding that additional research is needed in other sports in the aquatic environment, such as synchronized swimming [9].

Several studies show that the system of training synchronized swimmers should aim at complex approaches to motor capacity, through the systematic and scientific evaluation of the somatic and motor parameters involved in achieving performance goals [3,4,5,6,7,8,9,10,11,12]. Current trends in the training of synchronous swimming practitioners aim at a focus on specialized physical training, on improving elasticity, coordination, amplitude, and strength in relation to technical requirements. Also, the primary and secondary selection, as well as the training process in synchronous swimming must aim at respecting the age particularities aiming at the somatic and motor development. Scientific training in synchronous swimming aiming at the interrelationship between physical, technical, and artistic training is the premise for obtaining high sports performance.

In aquatic sports activities, physical training involves training in both the aquatic environment and in gymnasiums. Synchronized swimming requires athletes a higher level of development of motor qualities such as strength, endurance, and especially flexibility, which can be improved more effectively by exercising in various environments [13]. Studies have shown that flexibility in girls aged 12–14 years, shows significant differences between those who practice synchronized swimming and those who practice other types of water sports, concluding that the development of this motor skill is a consequence of the process of training in synchronized swimming and offers an advantage in obtaining successful results [14,15].

Taking into account the main positions specific to synchronized swimming, we made correspondence between the specific positions, the joints involved in the movement, and the required segmental flexibility (Table 1) The images were taken from the website of the synchronized swimming federation [8].

Studies on the aspects of flexibility in synchronized swimming, on different age categories are relatively few and focus mainly on classic mobility tests. We believe that studies should aim at a complex assessment of flexibility involving both classical tests and the degree of flexibility in performing figures specific to synchronized swimming in relation to the particularities of age and level of training.

The novelty of our study consists in evaluating the developed level of segmental flexibility in girls, by age categories for 7–14 years in relation to the level of sports training in synchronized swimming. This study can provide a concrete orientation of the evolution of the values of segmental flexibility in girls who practice synchronized swimming necessary for specialists in the field in selecting the design and implementation of training methodologies, as well as in their orientation in the initial and secondary sports selection.

This study aims to evaluate the effects of applying a program to a specific exercise program on the segmental flexibility of synchronous swimming athletes aged 7–14 years, divided into groups of 2 years each.

## 2. Materials and Methods

### 2.1. Experimental Design

The interventional study used the devices to measure the anthropometric parameters: thalliometer for investigating height (cm); Tanita BC 730 scale for monitoring body composition parameters, metal anthropometric band for arm span, centimeter, and flexometer for segmental flexibility assessment.

The present study was conducted in accordance with the guidelines of the Declaration of Helsinki, and it was approved by the Review Board of the Physical Education and Sports program, date of approval 3 May 2021. For this article, all authors contributed equally; also for this manuscript, all authors have an equal contribution with the first author.

Periodization of the study:

- SS1—group 7–8 years: initial testing 1–3 April 2021; implementation of the training program 5 April–3 June; final testing 5–7 June;
- SS1—group 9–10 years: initial testing 5–7 April 2021; implementation of the training program 8 April–7 June 2021; final testing 8–10 June 2021;
- SS1—group 11–12 years: initial testing 7–9 April 2021; implementation of the training program 12 April–10 June 2021; final testing 12–14 June 2021;
- SS1—group 13–14 years: initial testing 12–14 April 2021; implementation of the training program 15 April–14 June 2021, final testing 15–17 June 2021

All the tests and training programs were applied and practiced at the gym. All test data were collected by a trained team in a single session, which included anthropometric measurements followed by evaluation of physical parameters. Anthropometric measurements were performed before the start of the physical warm-up, at the beginning of the evaluation session. The physical tests were performed after a specific warm-up program lasting 20 min.

Anthropometric measurements included: Waist, Body Weight, and Span Arms, under similar conditions for all subjects [16]. The Span of the arms (SA) was measured using a metal anthropometric tape to assess the distance between the tip of the middle finger (finger) of the right hand and the tip of the middle finger of the left hand, standing with the arms sideways, the subject is positioned dorsally to a wall.

Segmental flexibility was assessed using 5 tests:

- Flexibility of the spine: standing, with legs, close together and the tips at the edge of the test apparatus (Digital Avant Flexometers), subjects make a maximum forward bending of the spine while maintaining this position for three seconds, record centimeters displayed digitally on the Flexometer;
- Flexibility of the hip and lower limb joints consisted of 3 tests: the maximum lateral distance of the legs (lateral string) in the horizontal plane, measuring centimeters from the pubis to the ground; the maximum antero-posterior distance in the horizontal plane of the legs (breaking) with the two variants: the right leg forward or the left leg forward, it is measured how many centimeters are from the pubis to the ground
- Shoulder flexibility: from a standing position with arms outstretched and crossed back, keep your arms crossed as far as possible and measure the distance between the middle fingers of the two palms.

### 2.2. Periodization of Training

The physical training specific to synchronized swimming consists of two workouts performed in the aquatic environment and a workout in the gym, for all groups of girls. The flexibility development program was performed during gym workouts, including exercises in acrobatic gymnastics, rhythmic gymnastics, and basic gymnastics, with an emphasis on improving the range of motion (Table 2). To the training performed in the swimming pool during the time spent in the water, according to the table above, warming on the ground is added on average of 20 min. The training program focused on developing flexibility included segmental gymnastics exercises adapted to the technical specifics of synchronized swimming. Each training lesson focused on a part of general muscle warm-up, followed by specialized training including stretching exercises, segmental gymnastics exercises performed with high amplitude, and at the end a part of muscle relaxation exercises. The training program had the same duration and weekly frequency for all research groups.

### 2.3. Subjects

This is an interventional study with a convenience sample, consisting of 112 girls aged between 7 and 14, who practice synchronized swimming at the Torpy sports club in Targu Mures, Romania. The subjects included in the study were divided into groups according to age, every two years, and the period of synchronized swimming. Parents or legal tutors, as well as the participant in the study, provided informed consent to participate in this study. The anthropometric features are presented in Table 3.

Sampling includes:

- SS1 group consisting of 32 girls aged 7–8 years, who practice synchronized swimming for 6–8 months, subjects participate in national competitions only as spectators;
- SS2 group consisting of 42 girls, aged between 9–10 years, with a sports experience of 12–18 months and who participates once a year in national competitions;
- SS3 group consisting of 28 girls, aged between 11–12 years, with a sports experience of 18–24 months and who participates twice a year in national competitions, as well as twice a year in interclub competitions;
- SS4 group consists of 30 girls, aged 13–14 years, with a sports experience of 24–42 months and who participate twice a year in national competitions and 2–3 international competitions per year.

Anthropometric results reveal a uniform development and within the limits of normality of all subjects of the experiment.

### 2.4. Statistical Analysis

In this study we used SPSS 24 software to calculate the following statistical parameters: arithmetic mean (X), standard deviation (SD), minimum (Min), maximum (Max), effect size, the statistical power of the sample (SP), Confidence Interval for the mean (CI–95%) with the two benchmarks lower and upper, Also, we calculate the Kolmogorov-Smirnov test to test the normality of data distribution.

For Cohen’s effect size interpretation, we used: over 0.20 means small effect size, over 0.50 means medium effect size, over 0.80 means large effect size. For this study, the significance was set at *p* < 0.05 for all analyses.

## 3. Results

The evolution of the development of segmental flexibility compared to the 4 age stages considered for the study was ascending with no periods of regression in terms of all segmental flexibility tests. Table 4 presented the descriptive results for all parameters for evaluating the flexibility of the 4 categories of subjects grouped by age.

The most significant confidentiality intervals of the initial test, per category of ages, were recorded at the Hip—split antero-posterior with the right foot forward for the 7–8-year-old group CI95% = 5.21–17.24; for the shoulder flexibility in the group of 9–10 years CI95% = 24.08–34.58; for the shoulder flexibility in the 11–12 age group CI95% = 24.93–37.95; for the hip—split antero-posterior with the right foot forward in the group of 13–14 years CI95% = 3.26–14.79. The most significant confidentiality intervals of the final test, per category of ages, were recorded at the shoulder flexibility for the 7–8-year-old group CI95% = 26.51–33.61; for the shoulder flexibility in the group of 9–10 years CI95% = 24.08–34.58; shoulder flexibility for the 11–12-year-old group CI95% = 24.35–35.07; for the hip—split antero-posterior with the right foot forward in the group of 13–14 years CI95% = 3.64–15.02.

The analysis of the results of the study in all tests and for all age categories reflects the fact that the values of the Cohen parameter were between 0.13 and 0.46 which reflects a small size of effect size. The values of statistical power were between 0.80 and 0.87, which shows that the values of the study are statistically significant compared to the threshold of 0.80 (Table 4).

**Figure 1:**
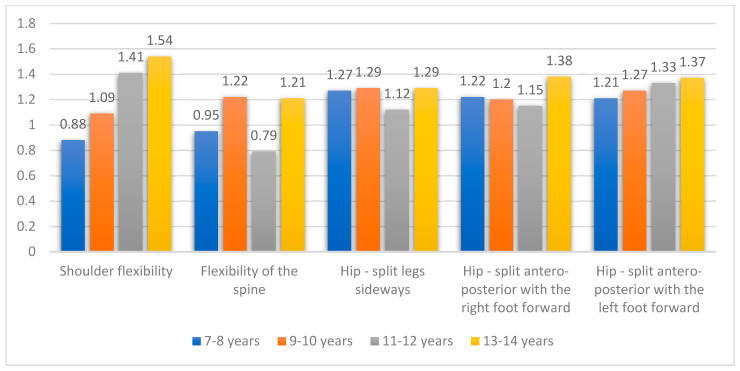
Evolution of segmental flexibilities related to the age groups.

The processing of the results showed that the segmental mobility varies depending on age, the segment involved in the movement, and the training period (Figure 1). For a better highlighting of the differences of the averages between the groups, we would also realize a centralization of them presented in Table 5. Analyzed the averages of the differences between final and initial tests we recorded: the biggest progress for shoulder flexibility was registered in the group of 13–14 years with 1.54 cm and the lowest in the group of 7–8 years with 0.88 cm; flexibility of the spine recorded the largest progress of 1.22 cm between the group of 9–10 years and the lowest progress with 0.79 cm for 11–12 years group; for hip—split legs sideways the biggest progress was recorded for groups of 9–10 years and 13–14 years with 1.29 cm and the lowest at 1.12 cm for 11–12 years group: the hip—split antero-posterior with the right foot forward showed the biggest progress between the group of 13–14 years with 1.38 cm and the lowest with 1.15 cm for 11–12 years group; the hip—split antero-posterior with the left foot forward showed the biggest progress between the group of 13–14 years with 1.37 cm and the lowest with 1.21 cm for 7–8 years group (Table 5). The study tests, for all groups, recorded values of the results of the Kolmogorov-Smirnov test showed good normality of the distribution of data recorded in the study for all age samples.

## 4. Discussion

Our study complements the level of general knowledge about synchronized swimming, and in particular, brings a scientific contribution on the impact of the level of training on the development of segmental flexibility, on 4 age categories at the junior level. The results of our study show that flexibility varies depending on age and implicitly depending on the level of sports experience. In synchronized swimming, sports training is differentiated on levels of training that aim at the minimum acquisition of a certain number of specific technical elements that require a high degree of flexibility and level of technicality. Flexibility has a determining role in optimizing the technical and performance level in synchronous swimming, and the comparison of the four age groups of junior athletes allowed us to identify the differences in evolution in developing the flexibility of different body joints involved in performing specific technical figures. The results of the study show a small size effect in all samples and for all age groups, which we consider to be the result of the limited period of time of only 3 months in which they had their specific intervention program to develop segmental flexibility.

The specific flexibility of synchronized swimming is very little studied, and previous research is mainly aimed at elite athletes, older than our subjects. On this premise, we will analyze the results of our study on flexibility compared to the results of other studies considering age. Flexibility is generally considered to be one of the most important motor skills, it involves the range of possibilities of movement of a single joint or a series of joints [17,18].

In the field of sports, several technical elements require a high degree of flexibility from athletes in various joints. Studies have shown that the higher the level of segmental flexibility, the better the level of performance in water sports, and synchronized swimming falls into this category [19,20].

The results of our tests are in line with the development trends of segmental flexibility identified in previous studies, so a study conducted in 2008 on passive and active balance flexibility on elite athletes with an average age of 20.6 ± 2.5 in the split legs sideways test, recorded the following results of the arithmetic mean of 2.82 (2.18), passive split and 1.77 (4.38) in an active split [21].

The flexibility of the spine favors the posture and the amplitude of the movement of the other body segments, being essential in performing the specific figures of synchronized swimming [22,23]. Our study highlights the importance of developing spinal mobility, being in line with previous studies. Thus, in a study performed on girls aged 8.60 ± 0.92, after an intervention program of 16 weeks of swimming, the progress of 16.79% was integrated. Also in this age category Sánchez-Rivas et al. (2014), on a group of 24 girls aged 7.84 ± 0.37, after a training program with a duration of 9 weeks, the frequency of 2 times per week, registered low progress of 0.6 ± 1.1cm, which means that improving flexibility in the 7–8 years category requires long periods of practice, and the results are moderate [24].

A comparative study was conducted on two age categories, aged 11–12 (n = 65) and 13–14 years (n = 70), female, aiming at the flexibility of the spine, between swimmers—experiment group and those who do not practice swimming, the results were the following: in the category 11–12 years 6.24 ± 4.19 in the control group, respectively 6.39 ± 4.93, and in the category 13–14 years the control group had 9.27 ± 10.04 and the swimmers 3.29 ± 14.3 [25]. These results are in line with the same trend demonstrated in our study.

Segmental gymnastics is an effective means of developing flexibility, at all ages, as evidenced by several studies [26,27,28,29]. A study of girls aged 9–10 years, with a segmental gymnastics intervention program twice a week, in physical education lessons, recorded a 0.3 cm retrogression in the mobility of the spine [30]. Another study aiming at developing the flexibility of the spine performed a sample of 49 girls aged 10.27 ± 0.31, recorded an average value at the initial test of 0.44 cm, and after the implementation of the 32-week program a final value of 1.30 cm [31], results being compared with our study. Similar results were recorded in studies that focused on the impact of segmental gymnastics on the flexibility of the spine or segmental to optimize technical and motor performance [32,33]. Studies have shown that synchronized swimming for young age groups, segmental flexibility can be improved by a specific operating methodology applied, being conditioned by periods of exercise [9,33,34,35].

Numerous studies highlight the interrelationship between sports experience and the content and duration of specialized training for the development of flexibility in relation to the vast range of practitioners [32,33,34,35]. The results of our study are relevant by highlighting the trend of developing segmental flexibility through tests specific to synchronized swimming, which can contribute to optimizing the contents of specialized physical training. The preoccupation of the specialists aiming at the development of flexibility from a young age represents the premise of the registration of superior performances from a physical and technical point of view [36,37,38].

Limits and strengths of the study. After completing the study, we were able to identify some relevant limitations. The study included only subjects aged 7 to 14 years. Extending the study to other age groups would help identify particular issues depending on the subject of the current study. In the study we considered only samples of girls; a relevant limitation of the current study was that the results of our study could not be compared with previous studies because their number is very limited and covers only some of the research aspects of us, which led us to refer to complementary sports such as artistic gymnastics and swimming. Another limitation would be the relatively small number of subjects included in the study, but this fact is due to the small number of junior girls’ practitioners of synchronized swimming in Romania. The flexibility tests of the spine also involve those of the hips and hamstrings, in our study we did not aim to evaluate the “Surface Arch Position” test, this fact we consider to be a limitation because not all tests were performed in immersion. We did not aim to compare the results of the research samples with control samples, because the number of practitioners on the vast categories studied is insufficient to correspond to the inclusion criteria.

The strengths of the study can be summarized as follows: the number of segmental flexibility parameters targeting the main segmental joints involved in performing technical elements in synchronized swimming, the complexity of specific tests to assess segmental mobility applied to junior synchronized swimmers; also the analysis on 4 age samples divided into groups every two years, framed in 7–14 years, the way of evaluating the impact of the specific training of synchronized swimming

## 5. Conclusions

The analysis of the research results between the age groups highlights the fact that between the initial and the final testing, the highest progress was registered for the flexible shoulder in the 13–14 year’s group, and the lowest progress at 7–8 year’s group; for the flexibility of spine the best result was obtained at 9–10 year’s group and the lower progress at 11–12 year’s group; for the hip-split legs sideways the best result was obtained at 9–10 and 13–14 year’s groups and the lower progress at 11–12 year’s group; for the hip-split antero-posterior with the right food forward the best result was obtained at 13–14 year’s group and the lower progress at 11–12 year’s group; for the hip-split antero-posterior with the left food forward the best result was obtained at 13–14 year’s group and the lower progress at 7–8 year’s group.

Following the analysis of the results, we can see an upward evolution of the development of joint flexibility for all age groups, and all joints considered in the test were in accordance with the particularities of growth and physical training development. The results of our study agree with previous studies on the level of development of segmental flexibility which is dependent on several factors including the age of onset of specialized training, the content of the training program that must be specialized for developing segmental flexibility in correlation with the specifics and particularities of the sport practiced and with the execution technique. The results of our study complete the level of knowledge on the development of segmental flexibility of synchronized swimming practitioners at the junior level, which may have a major future impact on the training methodology of athletes and implicitly on the level of development of the execution technique.

## Figures and Tables

**Table 1:** Correspondence between the movements performed, the joints involved in the movement, and the required segmental flexibility.

Position Name	Image	Involved Joint/Required Segmental Flexibility
Back Layout Position	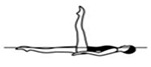	Coxo-femoral joint - hip flexibility
Flamingo Position	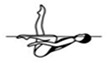	Coxo-femoral joint, knee Flexibility of the hip and legs
Ballet Leg Double Position	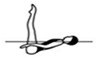	Coxo-femoral joint Balance flexibility
Crane Position	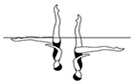	Coxo-femoral joint Balance flexibility
Tuck Position	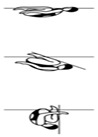	Coxo-femoral joint and knee Flexibility of the spine, hip, and legs
Front Pike Position	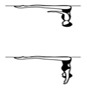	Coxo-femoral joint Balance flexibility
Back Pike Position	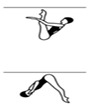	Coxo-femoral and scapulo-humeral joint Flexibility of the hip and spine
Surface Arch Position	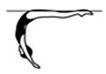	Hip, elbow, scapulo-humeral belt joint Flexibility of the spine, arms, neck
Bent Knee Back Layout Position	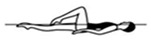	Knee and ankle joints Flexibility of the legs and hips
Split Position	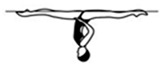	Coxo-femoral joint Flexibility of the hip and legs
Knight Position	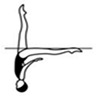	Coxo-femoral joint, spine, scapulo-humeral belt, and ankle Flexibility of the legs, hips, spine, and shoulder
Side Fishtail Position	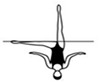	Coxo-femoral joint Flexibility of the hip, ankle

**Table 2:** Periodization of training by age categories.

Periodization	SS1	SS2	SS3	SS4
Months practice	3	3	3	3
Sessions of training per week/study	3/36	3/36	3/36	3/36
Minute of training per week in water	120	120	120	120
Minute of training per week in the gym	60	60	60	60

SS1—group 7–8 years, SS2—group 9–10 years, SS3—group 11–12 years, SS4—group 13–14 years.

**Table 3:** Anthropometric data (X ± SD) for the subject in the study.

Indicators	SS1	SS2	SS3	SS4
Number of subjects	32	42	28	30
Age (y)	7–8	9–10	11–12	13–14
Body mass (kg)	29.85 ± 7.61	33.55 ± 4.10	37.33 ± 5.96	49.76 ± 3.96
Stature (cm)	132.92 ± 9.57	140.97 ± 6.25	146.07 ± 4.78	160.36 ± 5.97
Body mass index (kg/m^2^)	16.89 ± 6.78	16.88 ± 4.98	17.49 ± 4.26	19.34 ± 4.01
Span Arm (cm)	130.06 ± 9.82	138.38 ± 8.82	145.60 ± 4.58	163.17 ± 6.65

SS1—group 7–8 years, SS2—group 9–10 years, SS3—group 11–12 years, SS4—group 13–14 years, X—mean, SD—standard deviation.

**Table 4:** Centralizer results in segmental mobility tests.

Group	Tests Description	Tests	X	SD	CI95% Lower	CI95% Upper	SP	Effect Size
SS1—group 7–8 years	Shoulder flexibility	TI	29.18	5.98	24.34	32.19	0.807	0.13
		TF	30.06	6.66	26.51	33.61		
	Flexibility of the spine	TI	7.11	5.12	5.11	9.87	0.812	0.14
		TF	8.06	5.09	5.34	10.77		
	Hip—split legs sideways	TI	−5.83	5.78	1.95	7.92	0.832	0.24
		TF	−4.56	5.26	1.86	7.25		
	Hip—split antero-posterior with the right foot forward	TI	−9.78	6.09	5.21	12.74	0.801	0.27
		TF	−8.56	6.54	5.07	12.04		
	Hip—split antero-posterior with the left foot forward	TI	−6.71	6.12	2.76	8.86	0.876	0.17
		TF	−5.50	5.47	2.58	8.41		
SS2—group 9–10 years	Shoulder flexibility	TI	28.62	12.32	24.08	34.58	0.811	0.20
		TF	29.71	11.77	24.35	35.07		
	Flexibility of the spine	TI	6.87	5.98	5.98	9.17	0.809	0.21
		TF	8.09	5.06	6.24	9.94		
	Hip—split legs sideways	TI	−11.76	8.79	5.68	13.76	0.834	0.23
		TF	−10.47	9.24	6.04	14.91		
	Hip—split antero-posterior with the right foot forward	Ti	−12.53	10.25	6.56	15.19	0.819	0.25
		TF	−11.33	9.78	7.10	15.55		
	Hip—split antero-posterior with the left foot forward	TI	−13.31	9.15	7.72	15.69	0.823	0.40
		TF	−12.04	8.85	8.01	16.07		
SS3—group 11–12 years	Shoulder flexibility	TI	30.12	11.72	24.65	37.85	0.872	0.46
		TF	31.53	11.42	24.93	38.13		
	Flexibility of the spine	TI	12.56	6.94	9.03	16.86	0.812	0.29
		TF	13.35	6.53	9.35	17.36		
	Hip—split legs sideways	TI	−11.59	6.81	5.09	12.11	0.804	0.22
		TF	−10.47	6.31	5.17	12.46		
	Hip—split antero-posterior with the right foot forward	TI	−8.97	6.77	3.92	10.76	0.827	0.23
		TF	−7.82	6.28	4.24	11.39		
	Hip—split antero-posterior with the left foot forward	TI	−9.54	5.91	5.13	10.84	0.831	0.25
		TF	−8.21	5.39	5.27	11.15		
SS4—group 13–14 years	Shoulder flexibility	TI	32.94	9.02	13.24	13.72	0.832	0.39
		TF	34.50	8.62	14.18	14.81		
	Flexibility of the spine	TI	15.22	6.82	12.21	18.92	0.810	0.46
		TF	16.43	6.56	12.79	20.07		
	Hip—split legs sideways	TI	−17.65	9.19	10.79	20.82	0.822	0.41
		TF	−16.36	8.88	11.45	21.28		
	Hip—split antero-posterior with the right foot forward	TI	−10.71	11.15	3.26	14.79	0.842	0.28
		TF	−9.33	10.67	3.64	15.02		
	Hip—split antero-posterior with the left foot forward	TI	−11.04	7.83	5.21	13.52	0.845	0.36
		TF	−9.67	7.45	5.54	13.79		

TI—initial test, TF—final test, X—arithmetic mean; SD—standard deviation, CI95%, Confidence interval for the mean, SP—statistical power, Effect size—Cohen’s effect size.

**Table 5:** Statistical description of differences of the averages of the groups between initial and final tests.

Group	Tests	DX	SD	t	t *p*_value_	K_S-Z	K-S *p*_value_
SS1—group 7–8 years	Shoulder flexibility	0.88	4.23	1.931	0.016	0.771	0.191
	Flexibility of the spine	0.95	5.71	1.762	0.013	0.655	0.085
	Hip—split legs sideways	1.27	4.11	2.20	0.034	1.247	0.089
	Hip—split antero-posterior with the right foot forward	1.22	3.75	2.064	0.024	1.004	0.265
	Hip—split antero-posterior with the left foot forward	1.21	3.91	2.764	0.009	1.534	0.081
SS2—group 9–10 years	Shoulder flexibility	1.09	4.12	1.867	0.041	0.621	0.135
	Flexibility of the spine	1.22	2.49	2.550	0.016	1.449	0.630
	Hip—split legs sideways	1.29	9.29	1.975	0.045	0.966	0.308
	Hip—split antero-posterior with the right foot forward	1.2	7.43	2.167	0.038	0.828	0.499
	Hip—split antero-posterior with the left foot forward	1.27	2.61	2.601	0.014	1.104	0.175
SS3—group 11–12 years	Shoulder flexibility	1.41	1.32	9.205	0.000	0.423	0.994
	Flexibility of the spine	0.79	1.27	3.499	0.002	0.628	0.825
	Hip—split legs sideways	1.12	3.17	4.259	0.019	0.897	0.297
	Hip—split antero-posterior with the right foot forward	1.15	2.98	1.360	0.021	1.230	0.197
	Hip—split antero-posterior with the left foot forward	1.33	2.31	2.724	0.011	0.846	0.472
SS4—group 13–14 years	Shoulder flexibility	1.54	1.56	3.453	0.001	0.342	0.418
	Flexibility of the spine	1.21	2.04	2.814	0.003	0.527	0.317
	Hip—split legs sideways	1.29	7.34	5.115	0.011	0.298	0.412
	Hip—split antero-posterior with the right foot forward	1.38	2.26	1.724	0.007	1.023	0.092
	Hip—split antero-posterior with the left foot forward	1.37	2.19	2.922	0.009	0.691	0.234

DX—a difference of arithmetic means between final and initial tests ((progress); SD—standard deviation, t—Student test, t *p*_value_—level of probability of Student test, K_S-Z—Kolmogorov-Smirnov Z test, K_S *p*_value_—level of probability of Kolmogorov-Smirnov Z test.
